# Factors that influence family and parental preferences and decision making for unscheduled paediatric healthcare – systematic review

**DOI:** 10.1186/s12913-020-05527-5

**Published:** 2020-07-17

**Authors:** E. Nicholson, T. McDonnell, A. De Brún, M. Barrett, G. Bury, C. Collins, C. Hensey, E. McAuliffe

**Affiliations:** 1 Centre for Interdisciplinary Research, Education and Innovation in Health Systems (IRIS) UCD School of Nursing, Midwifery and Health Systems, University College Dublin, Belfield, Dublin 4, Ireland; 2 Department of Emergency Medicine/National Children’s Research Centre, Children’s Health Ireland at Crumlin, Dublin, Ireland; 3 UCD School of Medicine, University College Dublin, Belfield, Dublin 4, Ireland; 4 Irish College of General Practitioners, 4/5 Lincoln Place, Dublin 2, Ireland; 5 Children’s Health Ireland at Temple St, Temple St, Rotunda, Dublin 1, Ireland

**Keywords:** Paediatric, Unscheduled healthcare, Decision-making, Preferences, Primary care, Emergency care, Out-of-hours

## Abstract

**Introduction:**

Health systems offer access to unscheduled care through numerous routes; however, it is typically provided by general practitioners (GPs), by emergency medicine doctors in in emergency departments (EDs) and by GPs in out-of-hours GP services such as practitioner cooperatives. Unscheduled healthcare constitutes a substantial portion of healthcare delivery. A systematic review was conducted to establish the factors that influence parents’ decision making when seeking unscheduled healthcare for their children. The systematic review question was **“**What are the factors that influence the decision making of parents and families seeking unscheduled paediatric healthcare?”

**Method:**

Five databases (CINAHL, PubMed, SCOPUS, PsycInfo, EconLit) and four grey literature databases (Proquest, Lenus, OpenGrey, Google Scholar) were searched. The titles and abstracts of 3746 articles were screened and full-text screening was performed on 177 of these articles. Fifty-six papers were selected for inclusion in the review. Data relating to different types of unscheduled health services (namely primary care, the emergency department and out-of-hours services) were extracted from these articles. A narrative approach was used to synthesise the extracted data.

**Results:**

Several factors were identified as influencing parental preferences and decision making when seeking unscheduled healthcare for their children. A number of the included studies identified pre-disposing factors such as race, ethnicity and socioeconomic status (SES) as impacting the healthcare-seeking behaviour of parents. Unscheduled healthcare use was often initiated by the parent’s perception that the child’s condition was urgent and their need for reassurance. The choice of unscheduled service was influenced by a myriad of factors such as: waiting times, availability of GP appointments, location of the ED, and the relationship that the parent or caregiver had with their GP.

**Conclusion:**

Policy and planning initiatives do not always reflect how patients negotiate the health system as a single entity with numerous entry points. Altering patients’ behaviour through public health initiatives that seek to improve, for instance, health literacy or reducing emergency hospital admissions through preventative primary care requires an understanding of the relative importance of factors that influence behaviour and decision making, and the interactions between these factors.

## Background

Unscheduled healthcare is healthcare which is typically provided in under 24 h’ notice [1]. Health systems offer access to unscheduled care through numerous routes; however, it is typically provided by general practitioners (GPs), emergency medicine physicians in emergency departments (EDs) and by GPs in out-of-hours GP services such as practitioner cooperatives. Unscheduled healthcare constitutes a substantial portion of healthcare delivery. From 1996 to 2010, 47.7% of hospital-associated healthcare was delivered in emergency departments in the USA [2] and attendances at emergency departments have been rising steadily at an international level [3]. Children are some of the heaviest users of unscheduled healthcare [1] and young children make up a large proportion of ED attendances that may have been treatable at primary care [4]. Moreover, in England, unplanned paediatric hospital admissions are also rising, particularly from EDs, and evidence has shown that these are typically short stays for minor conditions that, while often necessary for younger children, may be treatable in the community [5]. There are multiple complex factors and circumstances that can influence parents’ decision making regarding where, when and why they seek unscheduled healthcare for their children. At present, there is incomplete understanding of how parents and families make decisions when accessing unscheduled healthcare and the present review seeks to clarify this gap in the literature.

### Factors that influence care-seeking

As users of a health system, practical concerns, prior knowledge and experience of healthcare structures tend to feature heavily when patients are deciding where to attend unscheduled healthcare services, regardless of the urgency of the condition [6]. Such practicalities include availability of primary care appointments out-of-hours, advantages of the facilities available in the ED and the need for reassurance regarding their complaint [6]. Issues around access to and confidence in primary care are frequently cited as reasons for increased attendances at the ED, with the perceived urgency of the condition also noted [7–9]. Other pragmatic concerns that influence patients can be costs associated with certain services within the health systems and availability of transport to and from healthcare services [7]. Individual patient factors such as socioeconomic status (SES) can also dictate where care is sought with low SES typically associated with higher use of the ED [10–12], however, this is mediated by many complex interacting factors that require further exploration.

### Understanding the unscheduled healthcare system

Health systems must be adaptive to the needs of the populations they serve [13], and responding to evolving child health needs requires a systems approach to improve child health and access to health services in this population [14]. Access to healthcare is a complex interaction between the individual and the health system [15–17] and encapsulates the identification of healthcare needs through to the fulfilment of those needs by the health system [17]. The application of systems thinking can provide a pathway to addressing challenges within a health system [13], and evidence of the whole system is required in order to understand demand for its individual components and the complexities that govern how patients utilise them [7].

### A system of unscheduled healthcare

Much of the research in this area to date has focused on the use of primary care, ED and out-of-hours care as individual components within the health system or has focused on the use of scheduled or specialist health services. The variety of services that offer unscheduled healthcare has given rise to the argument that they should be studied as one system rather than as individual components within a health system [1, 18]. There is little evidence regarding the utilisation of unscheduled healthcare as a single system with multiple options that offer unplanned healthcare when needed, and critically, how patients make decisions about care seeking within that system. Many potential factors that influence where patients choose to seek care exist and accessibility and convenience also shape their care-seeking behaviour. This may result in a blurring of the boundaries between the types of unscheduled care on offer, which can be confusing for patients as they try to navigate this complex system and seek the most appropriate care [19]. When making such decisions, patients draw on existing knowledge about their health and the health system within a specific context [19]. Unpacking this process is critical to respond appropriately to healthcare needs. The aim of the current systematic review will seek to identify the factors that influence parents’ and families’ decision making when seeking unscheduled paediatric healthcare.

## Methods

### Review question

What are the factors that influence decision making of parents and families seeking unscheduled paediatric healthcare?

### Protocol and registration

The protocol for this systematic review was published on HRB Open Research [20] and was also registered on PROSPERO (ref: CRD42019129343).

### Search term identification

A preliminary search of PubMed and CINAHL using a limited number of key words was carried out to identify primary keywords used in the titles and abstracts of articles that will emerge in the search engines. These were used to formulate the search terms that were used in the systematic review.

### Timeframe

01/01/2000–12/03/2019.

### Key words

Keywords and Boolean operators are outlined in Table 1.

**Table 1:** Keywords and Boolean Operators

Child* OR paediatric OR pediatric OR Infant OR adolescent AND	
Parent* preferences OR choice* OR decision making OR Family preferences OR Reasons AND	
primary care OR general practice OR family physician OR emergency care OR emergency department OR out-of-hours OR Practitioner Cooperative OR after hours OR urgent care cent*	

* Indicates truncated search to capture words in American and British English

### Inclusion criteria

- Only studies published in English were considered for inclusion
- Empirical studies
- Studies that directly sought to establish factors that influence the decision making for the access of paediatric unscheduled healthcare (only factors that were explicitly reported from primary sources were included e.g., factors pertaining to socioeconomic status were not inferred from the data provided but had to be explicitly stated by authors)

### Exclusion criteria

- Studies that elicited factors that influence decision making for accessing adult healthcare or combined child/adult data
- Studies related to scheduled or specialist healthcare services
- Expert opinion or editorials
- Studies that used secondary data as the only data source (e.g., hospital administrative data)

### Databases

The 5 databases (CINAHL, PubMed, SCOPUS, PsycInfo, EconLit) were selected to capture a wide range of specialities and disciplines. A full electronic search of PubMed with limiters is provided in Supplementary Table 1.

### Grey literature

The search strategy described above was used to search ProQuest Dissertations and Theses, while modified search strategies were used to search Lenus, OpenGrey and Google Scholar. The results of the first 10 pages in Google Scholar were screened for inclusion in the study.

### Types of study to be included

All study types were included in the review provided they met the inclusion and exclusion criteria.

### Screening

**Fig. 1:**
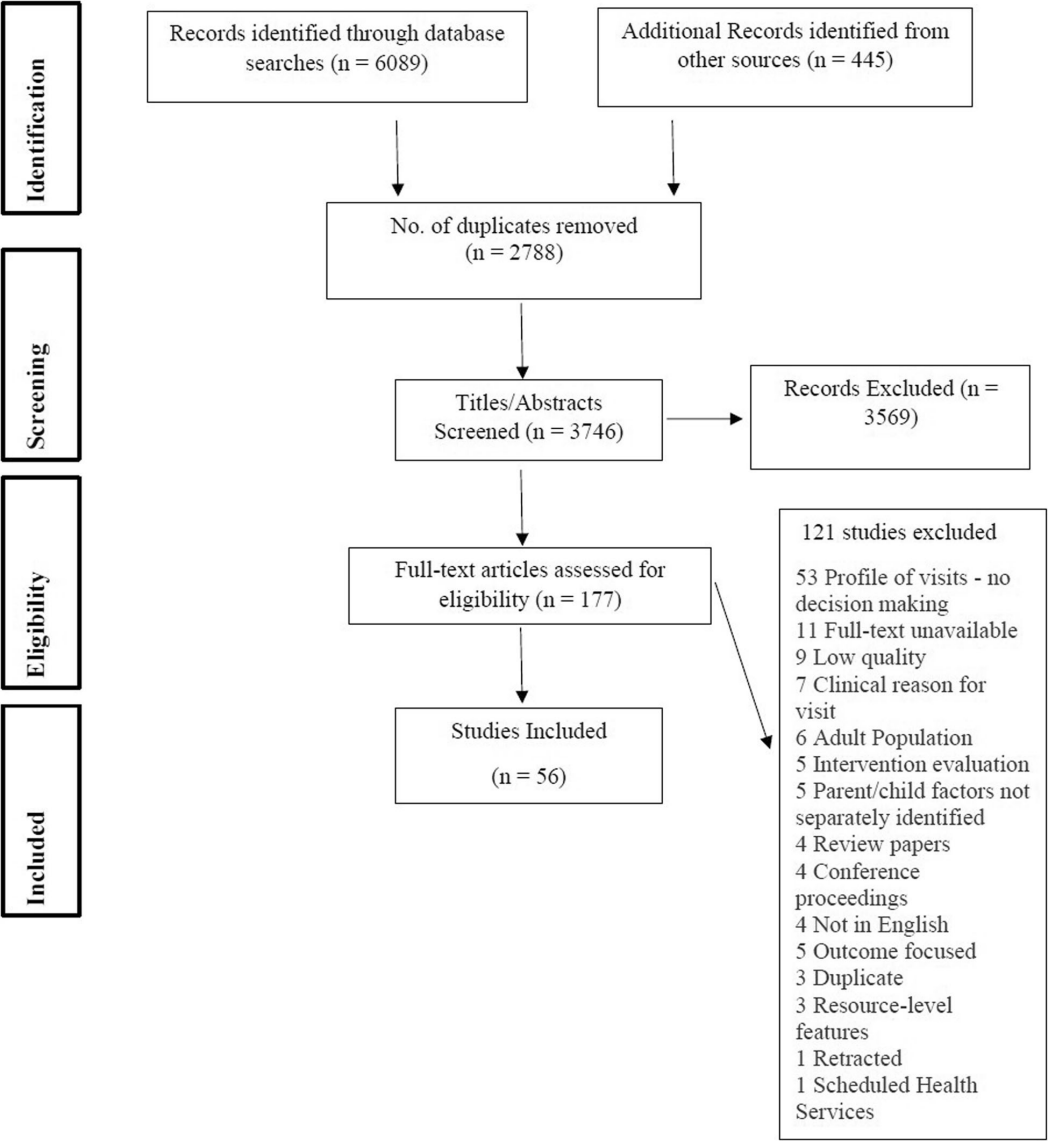
PRISMA Flow Diagram

Two authors independently screened the title and abstracts of search records retrieved against eligibility criteria. Full-text publications of all potentially relevant articles, selected by either author, were then retrieved and examined for eligibility. The search strategy and study selection is documented in the PRISMA flow diagram (Fig. 1) [21]. The reference list of each included article was also searched to identify additional relevant papers, and this yielded a further 9 articles for screening.

### Data management

The review management website Covidence™ [22] was used to remove duplicates and sort exclusions and inclusions using the create group function.

### Data extraction

Table 2 outlines the data that was extracted from the included studies. Three categories of data that were initially planned to be collected were covered by other fields (i.e., Research Question) or were not reported in the studies (e.g. details on health system, reasons for attendance). The data extracted includes general information related to the study, country of origin, and the aims and rationale of the research. Some variables (i.e., socioeconomic factors) were not consistently reported across the studies and any factors that were recorded were extracted (e.g., level of education, occupation etc.). With regards to the paediatric population in question, the relationship to the child (e.g., mother, father, caregiver), age, any disease groups or conditions was noted and the reason for attendance at unscheduled care, if reported. One reviewer extracted the data from the included studies and approximately 10% (*n* = 5) were checked for consistency by a second reviewer. There was 90% agreement rate between the two reviewers. Any discrepancies typically arose from a lack of clarity in the reporting of the papers and were discussed and agreed between the two reviewers. A narrative approach was used to synthesise the extracted data.

**Table 2:** Data Extraction Form

**General Information**	
Article Title	
Date	
Authors	
Country of Origin	
**Introduction**	
Aims and Rationale	
**Participant Details**	
Sample Size	
Age	
Gender	
Relationship to child	
Socioeconomic factors	
**Paediatric Population**	
Age	
Specific disease group or condition (if any)	
**Methods**	
Sampling Strategy	
Study Design	
Data Collection	
Data Analysis	
**Outcomes**	
Factors influencing behaviour and/or decision making/ Preferences elicited	

### Data availability and dealing with missing data

All data underlying the results were available as part of the article and no additional source data were required. There was no missing data in any of the included studies. The full text of 11 papers could not be accessed despite attempts to contact study authors for full texts using a maximum of three e-mails. After 3 weeks, if there was no response the review proceeded without these papers. Of the excluded papers, 7 were dissertations and 9 were from the USA with 1 from both the UK and Italy.

### Quality assessment

The Mixed-Methods Appraisal Tool (MMAT) [23] was used to assess the methodological quality of included studies. Papers selected for data extraction were evaluated by one reviewer (EN), prior to inclusion in the review. A second reviewer (TMcD) reviewed 25% of the studies to check for consistency. There was 93% agreement between the two reviewers with any disagreements resolved through discussion or consultation with a third reviewer (EM). The results of the quality assessment can be found in Supplementary Table 2. Given the large number of studies that emerged in the searches, those with a quality score of 25% or less were excluded from the review. Evidence from the literature has found that the exclusion of inadequately reported studies is unlikely to affect the overall findings of a review [24].

## Results

### Overview of included studies

A total of 56 published studies were included in the systematic review. Countries of origin included USA (*n* = 29), Australia (*n* = 10), Canada (*n* = 4), the UK (*n* = 5), Belgium (*n* = 1), The Netherlands (*n* = 2), Sweden (*n* = 1), Singapore (*n* = 1), Denmark (*n* = 1), Brazil (*n* = 1) and Lithuania (*n* = 1), which represented a broad array of health systems in the review. Few studies provided details on the health system where the research took place, however, the range of countries from which the included studies originated suggests a broad array of different health systems.

A range of methodologies emerged in the review with some utilising quantitative techniques such as surveys and questionnaires (*n* = 32), qualitative inquiry such as interviews and focus groups (*n* = 19), mixed-methods (*n* = 2), and discrete choice experiments (*n* = 3).

For studies that employed quantitative methods, the most common means of analysing data was through descriptive and other statistical analyses (odds ratios, modelling) (*n* = 31). The majority of qualitative studies used thematic analysis to analyse the data or generated common themes (*n* = 11), however, content analysis (*n* = 3), an iterative thematic approach (*n* = 1) and grounded theory (*n* = 4) were also used.

Regarding the ranges of ages represented in the paediatric samples in each particular study, they ranged from a minimum of 0–28 days, while the older cut-off ranged from 17 (the most common cut-off for a paediatric sample) to 18 in 3 studies [25–27]. Thirty-nine studies did not report the age of the paediatric sample. Table 3 outlines the demographics of the participants in each included study.

**Table 3:** Demographic details from included papers

Author & Date	Sample Size	Age & Gender of Caregivers	Relationship to Child	Age of Paediatric Population	Socioeconomic Factors	Specific disease group or condition (if any)
Albrecht et al., 2017 [28]	15	21–30 yrs. (20%), 31-40 yrs. (73%), 41–50 yrs. (7%); 80% female	Parent/caregiver	< 1 yrs.: 27%, 1–2 yrs.: 53%, 3–5 yrs.: 13%, > 5 yrs.: 7%	Highest level of education: Less than high school: (14%), High school diploma: (14%), Post-secondary certificate/diploma: (20%), Post-secondary degree (20%), Graduate degree (33%); Average household income: Under $25,000 (20%), $25,000–$49,000 (20%), $50,000–$74,999 (13%), $75,000–$99,999 (27%), $100,000–$149,999 (27%)	Vomiting & diarrhea
Augustine et al., 2016 [29]	13	Not reported	Parent/caregiver	Not reported	Not reported	Return visits
Bartlett et al., 2001 [30]	140	Mean = 33.3 yrs. (SD 6.7); 100% female	Mothers	Mean: 7.9 yrs. (SD 2.2)	70% completed high school or obtained a General Education Development certificate and reported having state-sponsored medical assistance (56%) or private health care insurance (36%).	Asthma
Benahmed et al., 2012 [31]	3117	Not reported	Parent/caregiver	Mean: 3.3 yrs	16.7% were considered disadvantaged	Non-urgent
Bernthal et al., 2017 [32]	31	Not reported	Parent/caregiver	Not reported	Not reported	Not reported
Berry et al., 2008 [33]	37	Mean: 28 yrs. Range:18–59 yrs.; 73% Female	Parent/caregiver	Mean: 3 Range:1.5–11 yrs.	68% public insurance, 18% private insurance	Not reported
Bingham et al., 2015 [34]	1531	Not reported	Parent/caregiver	Not reported	Not reported	Not reported
Buboltz et al., 2015 [35]	12	Not reported; 100% Female	Mothers (1 grandmother)	Not reported	Considered part of a vulnerable population	Not reported
Burokienė et al., 2017 [36]	381	< 35 yrs. (69.5%) Gender not reported	Parent/caregiver	Not reported	65% held a university degree	Non-urgent
Cabey et al., 2018 [37]	57	Mean: 33 yrs.; Gender not reported	Parent/caregiver	Mean: 6.5 years	56.1% employed; 28.1% private insurance	Not reported
Chin et al., 2006 [38]	12	Not reported	Parent/caregiver	Not reported	All were from zip codes known to represent low- income areas.	Not reported
Cooper et al., 2003 [39]	694	Not reported	Parent/caregiver	Not reported	Not reported	Not reported
Ellbrandt et al., 2018 [40]	657	Not reported	Parent/caregiver	Range: 0-17 yrs	Lower socioeconomic status contributed to direct care-seeking by almost 40% of parents	Not reported
Fieldston et al., 2012 [41]	25	Not reported	Parent/caregiver	< 5 yrs	Not reported	Not reported
Fredrickson et al., 2004 [42]	2773	Not reported	Parent/caregiver	Range: 0-17 yrs	Medicaid insured children included	Asthma
Freed et al., 2016 [43]	1150	Age Range: 20-40 yrs. Gender not reported	Parent/caregiver	< 9 yrs	Not reported	Lower urgency
Gafforini et al., 2016 [44]	1150	Age Range:20-40 yrs. Gender not reported	Parent/caregiver	< 9 yrs	Not reported	Lower urgency
Grant et al., 2010 [45]	112	Not reported	Parent/caregiver	Mean: 5.7 yrs	95% were African American and 5% white; 80.6% had Medicaid/SCHIP, 7.8% commercial, and 3.9% other insurance; 7.8% were uninsured	Not reported
Grigg et al., 2013 [46]	20	Range: 26-35 yrs. (65%); 79% Female	Parent/caregiver	Not reported	Latino families	Non-urgent
Guttman et al., 2003 [25]	331	Not reported	Parent/caregiver	Range: 0-18 yrs	45% private insurance	Non-urgent
Harrold et al., 2018 [47]	1533	Not reported	Parent/caregiver	Range: 0–28 days	Not reported	Not reported
Hendry et al., 2004 [48]	465	Not reported	Parent/caregiver		Lower socio-economic were over-represented	Not reported
Ingram et al., 2013 [49]	60	Age not reported 91% Female	Parent/caregiver	Range: 0-17 yrs	Stratified for socio-economic factors	Respiratory Infection
Janicke et al., 2003 [50]	87	Mean: 38.4 yrs. 94.3% Female	Parent/caregiver	Range: 4-9 yrs	White (89.7%), married (87.4%), and upper-middle socioeconomic status families. 96.6% had health insurance	Not reported
Klein et al., 2011 [51]	Interviewed (N ¼ 20) and non-interviewed (N ¼ 65)	Not reported	Parent/caregiver	Range:24-29mths	Mostly Medicaid insured	High frequency visitors
Kua et al., 2016 [52]	49	Age Not reported 55% Female	Parent/caregiver	Mean: 4.3 yrs., Range: 0-15 yrs	Not reported	Non-urgent
Kubicek et al., 2012 [53]	106	Not reported	Parent/caregiver		45% held an educational level lower than high school; 76% identifiedas Latino/Hispanic, 10% as African American, 7% as White/Caucasian and 5% as Asian; 49% reported an annual household income ofless than $20,000	Non-urgent
Lara et al., 2003 [54]	234	Mean: 31.5 yrs.; 80% Female	Parent/caregiver	Mean: 9.4 yrs	69% Latin American	Asthma
Lass et al., 2018 [55]	9	Range: 27-42 yrs.; 100% Female	Parent/caregiver	Range: 1-8 yrs	High-income sample	Not reported
Long et al., 2018 [26]	96	Range: 18-22 yrs.; Gender not reported	Parent/caregiver	Range: 0-18 yrs	91.7% had high school degree level of education. 60.4% had full-time jobs. 32.3% had an estimated annual income of $35,000 of estimated total annual household income.	Not reported
May et al., 2018 [56]	50	Range: 18-45 yrs.; Majority Female	Parent/caregiver	Range: 0-8 yrs	45–80% low income	Not reported
Morrison et al., 2014 [57]	299	Not reported	Parent/caregiver	Median: 2 yrs.	Not reported	Non-urgent
Mostajer et al., 2016 [58]	15	Not reported; 66% Female	Parent/caregiver	> 10 yrs	Not reported	Dental
Newcomb et al., 2005 [27]	403	Range: 14-19 yrs.;-50-63 yrs. Majority Female	Parent/caregiver	Range: 4mths-18 yrs	Publicly insured children	Non-urgent
Nokoff et al., 2014 [59]	234	Mean = 31.5 yrs.; 90% Female	Parent/caregiver	Mean: 5/6 yrs	Not clear	Acute illness
Ogilivie et al., 2016 [60]	337	Range: 24-45 yrs.; 79.5% Female	Parent/caregiver	< 18 yrs	4 deprivation deciles, Most deprived (19%)	Not reported
Pethe et al., 2019	120	Median = 4.5 yrs.; Gender not reported	Parent/caregiver	Range: 0-19 yrs.	Not reported	Not reported
Phelps et al., 2000 [61]	200	Mean = 30 yrs.; 82% Female	Parent/caregiver	Mean: 6.2 yrs	60% unemployed	Not reported
Philips et al., 2012 [62]	166	Mean = 31 yrs.; 76.5% Female	Parent/caregiver	Not reported	Not reported	Not reported
Philips et al., 2010 [63]	166	Mean = 31 yrs.; 76.5% Female	Parent/caregiver	Not reported	Not reported	Not reported
Salami et al., 2012 [64]	53	Not reported; 78.8% Female	Parent/caregiver	Not reported	Mostly Hispanic & African American	Not reported
Scott et al., 2003 [65]	3326	Mean = 34 yrs., Range: 16-75 yrs.; 87.4% Female	Parent/caregiver	Mean: 6.9 yrs. Range: 0–25 yrs.	17% University Educated	Not reported
Sharma et al., 2014	22	Not reported	Parent/caregiver	Range: 4mths - 12 yrs	Not reported	Not reported
Siminski et al., 2008 [66]	not reported	Not reported	Parent/caregiver	Range: 0-14 yrs	Not reported	Not reported
Smith et al., 2015 [67]	300	Not reported	92% parents	Range: 1–13 yrs	Not reported	Not reported
Stanley et al., 2007 [68]	422	Not reported	81% mothers, 12% fathers, 7% a grandparent/guardian.	Range: 0-17 yrs	51% Medicaid enrolees, 43% privately insured	Non-urgent
Stingone et al., 2005 [69]	5250	Not reported	Parent/caregiver	Range: 5 -12 yrs.	Not reported	Asthma
Stockwell et al., 2011 [70]	118	Not reported	Parent/caregiver	Mean: 6.4 yrs	Most children Latino, had Medicaid, in less than excellent health, had a foreign-born mother with a high school education or less	Influenza-like symptoms
Stoddart et al., 2006 [71]	15	Mean: 32.4 yrs. (exc.grandmother); 93% Female	Parent/caregiver/grandmother	Not reported	Not reported	Not reported
Turbitt et al., 2016 [72]	1146	Range: 20-40 yrs. Gender not reported	Parent/caregiver	Range: 1-9 yrs	13% had estimated annual income of less than $25,000	Not reported
Vaughn et al., 2012 [73]	57	Not reported	Parent/caregiver	Not reported	Latino population, 61% unemployed	Not reported
Williams et al., 2009 [74]	355	< 20 - > 40 years *N* = 349 Female	Parent/caregiver	Not reported	10.7% - Most disadvantaged 18.2% -Disadvantaged 70.1% -Least disadvantaged	Non-urgent
Woolfenden et al., 2000 [75]	25	Not reported	Parent/caregiver	Not reported	Not reported	Non-urgent
Zandieh et al., 2009 [76]	170	Not reported	Parent/caregiver	ED Mean: 7 yrs., PCP Mean: 5 yrs	Not reported	Non-urgent
Zickafoose et al., 2015 [77]	820	Range: 18-60 yrs.; 54% Female	Parent/caregiver	Range: 0-17 yrs	Not reported	Not reported
Zickafoose et al., 2013 [78]	20	Age not reported; 75% Female	Parent/caregiver	Range: 1 - 5 yrs	Not reported	Not reported

### Pre-disposing factors for use of unscheduled healthcare

A number of the studies included in the review identified pre-disposing factors such as race, ethnicity and socioeconomic status (SES) as having an influence in the care-seeking behaviour of parents. SES was typically reported using measures such as income, education, and deprivation level of the area where participants were living. The relationship between SES and child health is well documented [79], however, given the multi-faceted nature of SES, we only extracted data where SES measures were explicitly stated by the authors to be a factor that influenced attendance or care-seeking at unscheduled healthcare. SES interacts with factors such as race and ethnicity, which can also incorporate language and level of acculturation into the main culture [79]. These findings need to be balanced against the health system in which they occur with regards to local and structural issues and therefore, the countries in which such findings emerged are stated in Table 4.

**Table 4:** Study design, methods and factors that influence decision making

Author & Year	Country	Aims & Rationale	Study Design, Data Collection & Analysis	Sampling Strategy	Factors influencing behaviour and/or decision making/ Preferences elicited
Albrecht et al., 2017 [28]	Canada	Describe caregivers’ experiences of a child with pediatric AGE and to identify their information needs, preferences, and priorities.	Qualitative; Semi-Structured interviews; Thematic analysis	Qualitative	Child’s symptoms were perceived to not be improving fast enough, worsening symptoms, previous experience with a similar illness requiring emergency care, regular physician (i.e., family doctor or pediatrician) unavailable for consult, recommendation from another health provider (i.e., telephone health advice service, walk-in clinic doctor). Additionally, one caregiver was concerned that the child’s behaviour had changed, and another wanted to use the latest technology in the ED for the best diagnosis, management, and treatment.
Augustine et al., 2016 [29]	USA	Understand the reasons for pediatric return ED visits from the caretaker perspective.	Qualitative; focus groups; Thematic content analysis	Convenience sample of caretakers recruited by phone or in person at the return visit	Return visits occurred 12 to 50 h (median, 24 h) after the initial visit. Reasons for return visits were divided into 4 domains: (1) the caretaker’s response to the initial visit (e.g., dissatisfaction with medical staff, medical care, or information provided), (2) the child’s illness (e.g., continued, worsening, or concerning symptoms), (3) the nature of the ED itself (e.g., subspecialist availability, convenient hours), and (4) follow-up care (e.g., lack of appointments with primary physicians or subspecialists).
Bartlett et al., 2001 [30]	USA	Examine whether maternal depressive symptoms are associated with ED use.	Quantitative; Survey; Descriptive & Inferential Statistics	Children whose mothers reported that they had (1) asthma diagnosed by a physician, (2) day or night asthma symptoms, including wheezing, shortness of breath, and/or a cough at least once a week during the past 2 weeks, and/or (3) at least 1 visit for asthma to the ED in the previous 6 months or 1 overnight hospitalization for asthma in the previous year.	Mothers who reported the highest tertile of depressive symptoms also reported the most frequent use of the ED (Mantel- Haenszel test, ^2^ = 6.33, *P* = .01).
Benahmed et al., 2012 [31]	Belgium	Evaluate the rate of pediatric non-urgent use in ED in a subset of 12 Belgian hospitals and to determine the associated factors. The identification of such factors would help the policy marker to design a cost-effective pediatric care system.	Quantitative; Administrative data & questionnaire; Descriptive & multivariate statistics	Children who attended an ED of the 12 hospitals during the 2-weeks period.	Among the 3117 children, attending ED, 39.9% (1244) of visits were considered inappropriate. Five factors were significantly associated with inappropriate use: age of child, distance to ED, having a registered family doctor, out-of-hours visit, and geographic region
Bernthal et al., 2017 [32]	UK	Describe the impact of being a lone parent, particularly when fearful for their partner’s safety and the coping strategies employed by Army parents to combat the challenges presented by Army life.	Qualitative; Focus groups and interviews: observational prospective survey; Thematic analysis	Parents living or working within the garrison for the subsequent 3-month period	Making sense of the illness, knowing their child, fear for their husband’s safety and the impact of being a lone parent all influenced their decision making when their child was unwell. The mothers in this study found making decisions alone very stressful, particularly when the fear for their partner’s safety made them feel particularly emotionally vulnerable.
Berry et al., 2008 [33]	USA	Identify parents’ reasons for choosing the ED over primary care for non-urgent pediatric care through in-depth parental interviews.	Qualitative; Ethnographic interviews; Thematic analysis	Parents whose children had been to the ED for care during hours when physicians’ offices are routinely open (Monday to Friday, 8 AM to 4 PM) and assigned a nurse triage level of 5 on a 1 to 5 scale (with 5 indicating “no resources likely to be utilized,” or a non- urgent problem).	Parents bring children to the ED for non-urgent care during regular office hours because of PCP referral, better efficiency in the ED, dissatisfaction with PCPs, perceived higher quality of care in the ED, long waits to see PCPs, and PCP communication problems.
Bingham et al., 2015 [34]	Australia	Assess parents’ perspectives on the potential impact of co-payments for general practice and emergency department (ED) services for children.	Quantitative; Survey; Descriptive statistics & logistic regression	Parents of children presenting with lower urgency conditions (triage category 3, 4 or 5) to the EDs of three public general hospitals and one paediatric hospital in Melbourne	73% (*n* = 1089) of parents reported a $7 general practice co-payment would not increase their use of EDs for lower urgency problems for their children. Increased use was associated with younger parent or guardian age and lower household income.
Buboltz et al., 2015 [35]	Brazil	Understand the strategies of families in search of health care for children attended in pediatric first aid.	Qualitative; Semi-Structured interviews; Content analysis	Family caregivers of children who received care from the health team at the unit, selected based on the children’s medical histories	Caregivers’ used the private health system as a strategy to seek care when the public system was unavailable
Burokienė et al., 2017 [36]	Lithuania	Determine the factors influencing the parental decision to bring their child to the ED for a mi- nor illness that could be managed in a primary healthcare setting	Quantitative; Survey; Descriptive statistics	Purposive	Parents who brought their children to the ED without physician referral were five times more likely to visit the ED during evening hours and on weekends (OR = 5.416; 95% CI, 3.259–8.99; *p* < 0.001). The decision to come to the ED without visiting a primary care physician was made more often by parents with a higher income (OR = 2.153; 95% CI, 1.167–3.97) and those who came due to children having rash (OR = 4.303; 95% CI, 1.089–16.995) or fever (OR = 3.463; 95% CI, 1.01–11.876). Older parents were 2.07 (95% CI, 1.1224–3.506) times more likely to evaluate their child’s health unfavourably than younger parents.
Cabey et al., 2018 [37]	USA	Explore caretaker decision making processes, values, and priorities when deciding to seek care.	Qualitative; Interviews; Grounded theory	Purposively sampled patients with high or low frequency ED and primary care use for low-acuity visits.	Caretakers who used the ED frequently had limited social support and reported difficulty accessing care when compared to other caretakers. Fear also motivated care seeking and a desire for immediate medical care.
Chin et al., 2006 [38]	USA	Understand patterns of decision making among families presenting to a pediatric emergency department (ED) for non-acute care and to understand pediatric ED staff responses.	Qualitative; in-depth interviews, direct observations, and non-identifying demographic data; thematic	Children registered for care in the shifts under study during the 3-day study period.	Three main themes: [1] most families had been referred by their primary care providers [2]; the complexity of living in low-income areas makes the ED a choice of convenience for these stressed families; and [3] mistrust of primary health services was not identified as a motivator for ED utilization, in contrast with other published data.
Cooper et al., 2003 [39]	Australia	Identify parental reasons for presenting their child to the emergency department and their expectations of the consultation	Quantitative; Cross-sectional survey; Descriptive statistics & odds ratios	Parents of children and adolescents aged ≤14 years who presented to the Fairfield ED over a 2-month period	The majority of presentations were self-referred and chosen because of proximity. Majority of children do not require admission; however, parents often have expectations that observation and further investigation will occur prior to discharge from the emergency department.
Ellbrandt et al., 2018 [40]	Sweden	Evaluate the care-seeking patterns, availability of medical options and initial medical assessments – with overall reference to socioeconomic status – of parents at an urban paediatric emergency department in a Scandinavian country providing free paediatric healthcare	Quantitative, Questionnaire; Descriptive statistics	Children aged 0–17 years and arriving unscheduled at the paediatric ED during the day (0800 to 1659 h) and evening (1700 to 2059 h) and assessed physician during a peak period of 25 consecutive days in February and March	79% of parents either failed to or managed to establish medical contact before the emergency department visit, whereas 21% sought care with no attempt at recent medical contact. Visits with a failed telephone or primary care contact (18%) were more common outside office hours and were scored as less urgent. A perceived emergency was the main reason for no attempt at medical contact before the visit. Direct emergency department care-seeking was more common from the city district with the lowest socioeconomic status.
Fieldston et al., 2012 [41]	USA	Elicit and to describe guardians’ and health professionals’ opinions on reasons for non-urgent pediatric ED visits.	Qualitative; Focus groups; Thematic analysis		Perceptions of need for medical care or concern about severity of illness, systems issues, such as accessibility and availability of appointments, and some personal or family issues. Many guardians stated a need to receive timely reassurance about their concerns, particularly the more worried they were about the symptoms
Fredrickson et al., 2004 [42]	USA	Clarify the reasons for frequent ER use by Medicaid-insured children with asthma living in rural areas and 23 towns in Kansas as a first step in identifying primary care activities with the potential to reduce such use.	Mixed methods, Administrative data and focus groups; Descriptive Statistics	Medicaid-insured children and children with one or more hospitalizations or at least 3 ER visits for asthma	The decision to use ER services for childhood asthma in the Medicaid-insured population was driven by problems in using primary care services.
Freed et al., 2016 [43]	Australia	Determine why parents seek ED care for their child for lower urgency conditions	Quantitative; Survey; Statistics	Parents or guardians presenting to the ED with children	43% of parents attempted to make an appointment with a general practitioner (GP) for their child prior to presenting to the ED. Two-thirds of those who did contact a GP were instructed to go to the ED for their lower urgency condition. Few attempted to contact a nurse telephone triage service or after-hours GP service.
Gafforini et al., 2016 [44]	Australia	Assess parental preferences and experiences regarding the treatment of lower urgency child injuries and the role of general practitioners (GPs) in such care	Quantitative; Survey; Statistics	Parents or guardians presenting to the ED with children	Fewer parents of injured children, compared with illness, attempted to make a GP appointment prior to attending ED (35% vs 46%; P < 0.001). A greater proportion of injured children were referred to the ED by their GP than ill children.
Grant et al., 2010 [45]	USA	Explore reasons for non-urgent pediatric emergency department use in the Mississippi Delta	Quantitative; Interviews	Convenience	Only 24.3% tried to obtain care before emergency department visit; 23.2% said their children required “urgent” care. Mean distance from home to usual source of care was 10 miles. 10% cited transportation as a barrier to keeping health care appointments; 5.5% cited insurance or cost. Families who used the emergency department during evening/weekends were significantly more likely to have cited clinic hours of operation as a reason care was not sought previously than were “business hours” users, who emphasized convenience.
Grigg et al., 2013 [46]	USA	Investigate Latino parents’ decision to seek pediatric emergency care for non-urgent health conditions.	Qualitative; Focus groups; Grounded theory	Purposive	Parents were deeply concerned about the child’s fever, often giving acetaminophen but then seeking medical care when the fever returned. Avoiding double wait times was an important determinant of seeking care in the ED. As patients routinely had long waits to be seen in the clinic, the idea that one might have to “wait twice”—once in clinic and then again at the hospital—made parents more likely to seek ED care directly. Some parents found it particularly hard to obtain same-day clinic appointments for an acute illness. Participants were pleased with the quality of ED care.
Guttman et al., 2003 [25]	USA	Identify reasons for medically non-urgent ED visits from the users’ perspective	Qualitative; Interviews; Thematic approach	parents or guardians who came to the ED for a pediatric visit considered medically non-urgent by the ED triage staff	Caretakers said they came to the ED because it was important to get reassurance that the child’s situation was not serious or would not deteriorate
Harrold et al., 2018 [47]	Canada	Characterize neonatal visits to the emergency department and families to identify potential strategies to decrease neonatal emergency department visits.	Quantitative; Survey; Descriptive Statistics & correlational analysis	Convenience	The majority of respondents (73.9% [1104/1494]) had received advice before going to the emergency department. In most cases (86.4% [954/1104]), this was from a health care provider, who frequently advised going to the emergency department
Hendry et al., 2004 [48]	UK	Gather information on children with minor illness or injury presenting to a paediatric accident and emergency (A&E) department and the decision making process leading to their attendance	Quantitative; Survey; Descriptive statistics	New attenders to the paediatric A&E department during three survey periods	Educational attainment, childcare experience, and parental coping skills were important in relation to A&E attendance. More children attended with injury as opposed to illness. There were no significant demographic differences between those children who presented directly to A&E and those who made prior contact with a GP. Just under half had made contact with a general practitioner (GP) before attending A&E. The majority of those children were directly referred to A&E at that point. GPs referred equivalent numbers of children with illness and injury.
Ingram et al., 2013 [49]	UK	Explore parents’ views on support and information needs prior to consulting when children have RTIs with cough, and identify the triggers and barriers to consulting primary care	Qualitative; Focus groups and interviews; Thematic analysis	Purposive: identified through a search of patient records, in six GP practices, for those who had consulted in the previous 3 months for a child with a respiratory infection	The perception of threat to a child of RTI (with cough) was increased with more severe illness and by perceived susceptibility to illness of a particular child; whilst experience with other children increased parental efficacy to cope with childhood cough at home. Psychological models of health behaviour informed the understanding of cultural beliefs and attitudes that underpin health related behaviours.
Janicke et al., 2003 [50]	USA	Test social-cognitive influences on parent decision making processes related to children’s health care use.	Quantitative; Questionnaires, Statistics	Primary caretakers of children ages 4 to 9 years and their child	The best predictive model, accounting for 29.8% of the variance in primary care use, included the interaction between parental stress and self-efficacy to cope with parenting demands, child behavior problems, self-efficacy for accessing physician assistance, medication use, and parent health care use
Klein et al., 2011 [51]	USA	Determine the distribution and frequency of visits families make to a pediatric primary clinic; and to explore the reasons for families with frequent visits.	Mixed methods; Interviews and survey, Descriptive statistics and thematic analysis	High frequent attenders identified	Overall clinic environment, appointment availability, convenience, insurance/Medicaid, reputation, and friendly office staff.
Kua et al., 2016 [52]	Singapore	Understand the reasons behind non urgent ED visits, in order to develop targeted and effective preventive interventions	Qualitative; Interviews; Grounded theory	Caregivers of children who had been diagnosed with typical non-urgent conditions, namely fever, nosebleed and minor head injury, by the attending physician in the ED	Caregivers heavily influenced by the perceived severity of the disease in the child when deciding on where to go for medical care
Kubicek et al., 2012 [53]	USA	Develop a descriptive profile of parents and caregivers who bring their children to the emergency department for non-urgent issues	Quantitative; Survey; Descriptive statistics & thematic approach	Targeted purposive sampling	The majority of respondents described themselves as Latino (76%) and foreign-born (62%). About half (49%) reported having an annual income of less than $20,000 and 43% of respondents did not have health insurance for themselves. Almost all (95%) of the index children had a primary care physician and health insurance. In spite of being triaged as non-urgent, over half (63%) described their child’s condition as “very” or “extremely” urgent. About half of the respondents reported not receiving basic information on childhood illnesses from their child’s doctor. Reasons for non-urgent visits seemed to revolve around issues of convenience and perception of quality of care.
Lara et al., 2003 [54]	USA	Explore, in a predominantly Latino inner-city population, why caregivers bring their children with asthma to the ED (emergency department).	Quantitative; Survey & medical chart review; Descriptive statistics & thematic approach	Not clear	75% of caregivers cited worsening symptoms as the most important reason for bringing the child to the ED. 25% of parents reported bringing the child to the ED because they could not pay for care or another doctor or another clinic was inconvenient.
Lass et al., 2018 [55]	Denmark	Explore parental contact pattern to OOH services and to explore parents’ experiences with managing their children’s acute health problems.	Qualitative; Interviews; Inductive content analysis	Parents of children under age 4 recruited from a child day care centre in Aarhus, Denmark	Navigation, information, parental worry and parental development appeared to have an impact on OOH services use. The parents found it easy to navigate the health care system, but often used the OOH service instead of their own general practitioner (GP) due to more compatible opening hours and insecurity about the urgency of symptoms.
Long et al., 2018 [26]	USA	Determine which factors influence parents or guardians to choose the ED over their primary care physician (PCP)	Quantitative; Surveys; Descriptive statistics & Thematic approach	Parents or guardians of low-acuity pediatric patients.	Most patients had an established PCP. More than two-thirds did not attempt to contact their PCP prior to their ED visit. Nearly half stated their PCP did not offer after-hours or weekend availability. Most did not feel their child’s condition was serious. Almost half would have waited to see their PCP if they could be seen within 24 h
May et al., 2018 [56]	USA	Explore the decision to seek care and decision- making regarding location of care among parents with low and adequate health literacy.	Qualitative; Semi-structured interviews; Grounded theory	Purposive	Parents with low health literacy were more inclined to overestimate severity of illness and seek care sooner to gain answers about the illness and treatment options and visit the clinic only when an appointment was available within hours. Parents with adequate health literacy sought reassurance for their ongoing illness management and valued close relationships with their physician and were willing to wait longer for an appointment. Fever, vomiting, and young child age prompted some parents to seek expedient care regardless of health literacy.
Morrison et al., 2014 [57]	USA	Examine the association between caregiver health literacy and the likelihood of a non-urgent emergency department (ED) visit in children presenting for fever.	Quantitative; Questionnaire; Statistics	Purposive	Low health literacy was associated with a higher proportion of non-urgent ED visits (44% vs. 31%; OR 1.8; 95% CI 1.1, 2.9). Caregiver black race and public insurance were also related to non-urgent ED use in unadjusted analyses.
Mostajer et al., 2016 [58]	Canada	Explore the reasons that lead parents to select the ED over a dental clinic for their child’s non traumatic dental problem.	Qualitative; Semi-structured interviews; Thematic analysis	Parents of children under age 10 who sought care for non-traumatic dental problems in an ED of a pediatric hospital	Three themes emerged (i) Parental beliefs and socioeconomic challenges contributed to their care seeking, (ii) parents faced barriers in finding oral healthcare options for their children in their communities (e.g., poor access to care and poor quality of care), and (iii) parent’s high satisfaction with the care provided through the ED.
Newcomb et al., 2005 [27]	USA	Account for multiple factors in family decision making, including factors that have been speculative in the literature, but not specifically included together in other studies	Quantitative; Cross-sectional Survey; Descriptive statistics	Purposive	Access to primary care influenced their decision to seek care in the emergency room, as well as workload and quality problems at the primary care level
Nokoff et al., 2014 [59]	USA	Understand and compare caregivers’ perceptions of and attitudes toward care received in a primary care clinic (PCC) versus that received in the pediatric emergency department (PED) as well as the reasons for selecting either location to receive care for their child.	Quantitative; Survey; Descriptive statistics & odds ratios	Parents who brought their child (younger than 18 years) in for a sick visit	Compared with caregivers who brought their child to the PED, those who presented to the PCC were more likely to report that the child had been sick for more than 2 days (P G 0.001), indicate that the child could wait more than 3 h to be seen (P G 0.001), have called the PCC for advice (odds ratio [OR], 5.2; 95% confidence interval [CI], 2.9Y9.2), have spoken with a nurse (OR, 3.7; 95% CI, 2.0Y6.7), be satisfied with their phone call to the PCC (OR, 12.2; 95% CI, 6.4Y23.1), and report that they could easily get in touch with the PCC (OR, 3.6; 95% CI, 1.8Y7.3). Most caregivers who went to the PCC felt that it was more convenient (98.6%) and they would be seen more quickly (95.8%).
Ogilivie et al., 2016 [60]	UK	Understand decision making when bringing a child to an emergency department.	Quantitative; Cross-sectional Survey; Descriptive statistics	Parents attending the emergency department from 10:00 to 22:00, with a child aged 18	Younger parents reported feeling more stressed. Parents of younger children perceived the injury/illness to be more serious, reporting greater levels of worry, stress, helplessness and upset and less confidence.
Pethe et al., 2019	USA	Examine parental reasons associated with the decision to seek ED care in a group of low- income, inner-city, publicly insured children.	Quantitative; Survey; Descriptive statistics	Not clear	There was no difference in those who were aware of walk-in hours or an after-hours phone line and a reported ED visit. Half of the parents (52.5%) thought their child’s medical problem was serious.
Phelps et al., 2000 [61]	USA	Identify specific caretaker and utilization characteristics predictive of the use of the emergency departments (EDs) for non-urgent reasons	Quantitative; Questionnaire- descriptive study; Descriptive statistics	Caretakers who brought their children to 1 of 2 urban hospital EDs	Caretakers who reported being taken to the ED when they were children and those with Medicaid insurance were more likely to view the ED as the usual site of care. Being a single parent was a predictor for non-urgent visits
Philips et al., 2012 [62]	The Netherlands	Reveal the crucial decision criteria of patients in choosing out-of-hours services	Quantitative; Discrete Choice Experiment; Multinomial Logit Model	All consumers at the Free Newborn and Child health care service (FNC service) in Antwerp.	Patients considered the ‘explanation’ about the problem and the treatment as the most important factor in the choice of service (‘child’: 38.5%), followed by the waiting time for consultation (‘waiting time’: ‘child’: 23.8%).
Philips et al., 2010 [63]	The Netherlands	Identify consumers’ preferences for after-hours medical care and predict the use of the new GPDS.	Quantitative; Questionnaire, Statistics	All consumers at the Free Newborn and Child health care service (FNC service) in Antwerp.	Main reasons for choosing ED are “sufficient explanation” and “easy access”. Consumers also expect immediate technical examination at the ED and when visiting a paediatrician. Compared to the ED and the paediatrician, “waiting time” was the most appreciated attribute at the GPDS.
Salami et al., 2012 [64]	USA	Determine the most important reasons for pediatric non-urgent (NU) emergency department (ED) visits as perceived by caregivers,	Quantitative; Survey; Descriptive statistics	Convenience sample of low acuity visits (triage categories 4 and 5).	The reasons most important to the caregivers were “outside PCP working hours,” “lack of health insurance,” and “better hospitality in ED”
Scott et al., 2003 [65]	UK	Elicit the preferences of patients and the community for different models of GP out of hours care.	Quantitative; DCE; Random effects model	Parents of children in Aberdeen and Glasgow who had received a home visit or attended a primary care emergency centre, or were registered with a GP	The most important attribute was whether the doctor seemed to listen, suggesting that policies aimed at improving doctor–patient communication will lead to the largest improvements in utility. The most preferred location of care was a hospital accident and emergency department.
Sharma et al., 2014	Australia	Explore the reasons prompting Australian parents to seek medical advice for their sick children, and to define the factors influencing their decision.	Qualitative; Semi-Structured interviews; Thematic Analysis	Not clear	Five emergent themes were fears about possible scenarios; personal and vicarious experiences; resources and convenience; being seen to do the right thing; and reassurance and guidance about management.
Siminski et al., 2008 [66]	Australia		Quantitative; Survey; Descriptive statistics	Convenience	Problem too urgent, problem too serious, better service at ED
Smith et al., 2015 [67]	Canada	Explore the factors associated with parents’ decisions to bring their children to the pediatric emergency department (PED) for non-emergent concerns.	Quantitative; Cross- sectional survey; Descriptive statistics	participants who had contacted any health care provider (primary care physician [PCP], walk-in clinic, BC Nurse Line, another ED, or other) in the 48 h prior to coming to the PED	The top 3 reasons for coming to the British Columbia Children’s Hospital PED were (1) that it specializes in children, (2) child has medical issues previously managed at the same hospital, and (3) closest location to patient.
Stanley et al., 2007 [68]	USA	Explore parental rationale and the appropriateness of children’s visits to emergency departments (EDs) for non-urgent complaints.	Quantitative; Semi-structured interviews/ survey; Descriptive statistics	Parents/guardians of children aged 6 months to 18 years who presented to the ED with non-urgent complaints	The most common parent-reported reason for going to the ED was reassurance (41%), followed by thinking the situation was an emergency (33%).
Stingone et al., 2005 [69]	USA	Evaluate the role of socioeconomic, disease-related, and access-to-care factors in utilization of the ED and inpatient services for urgent treatment of asthma.	Quantitative; Cross - sectional questionnaire; Descriptive statistics	Schools were randomly selected based on the childhood asthma hospitalization rate in each neighbourhood.	In univariate analysis, use of urgent care was strongly associated with race/ethnicity and income
Stockwell et al., 2011 [70]	USA	Understand the utilization of the pediatric emergency department (PED) of an academic hospital during regular primary care office hours during the 2009 H1N1 epidemic.	Quantitative; cross- sectional survey/ secondary analysis; Descriptive statistics	Parents visiting a PED in a low-income area in New York City	No sociodemographic differences among children brought to the PED for ILI and those brought for other presenting symptoms
Stoddart et al., 2006 [71]	Australia	Design a qualitative pilot study aiming to explore this issue using semi-structured interviews	Qualitative; Semi-structured interviews; Iterative thematic approach	Parents attending GP	Parents sought an examination of their child (in particular “hidden areas” such as ears and throat) and reassurance, rather than antibiotics. They also wanted the GP to suggest practical ways to help alleviate their child’s symptoms.
Turbitt et al., 2016 [72]	Australia	Study the prevalence of a regular source of primary care for Victorian children attending one of four emergency departments (EDs) and to determine associated characteristics, including ED use.	Quantitative; Survey; Descriptive statistics	Parents or guardians of patients (≤9 years of age) attending the ED at one of four Victorian hospitals	No associations were observed between having a regular source of primary care and frequency of ED attendance in the past 12 months, although parents whose child did not have a regular source of primary care were more likely to view the ED as a more convenient place to receive care than the primary care provider
Vaughn et al., 2012 [73]	USA	Assess Latino immigrant usage, access, and reason for coming to the pediatric emergency department (PED) and clarify parental perceptions, barriers, and concerns regarding Latino children’s health.	Quantitative; Interviews & Survey; Descriptive statistics	Convenience	Latinos with lower levels of acculturation were more likely to use the PED to meet their children’s health care needs.
Williams et al., 2009 [74]	Australia	The primary aim of this study was to provide a comprehensive, systematic understanding of the motivations and actions of parents of children with non-urgent illnesses who attend a PED	Quantitative; Survey; Descriptive statistics	Parents of children who attended the PED with a non- urgent condition	The factors identified were: parents rated their child’s condition as moderate to very serious (242 (68%)); two-thirds of parents (234 (66%)) had sought advice prior to attending the emergency department; 54% [68] of the 137 children who attended with an injury presented promptly to emergency (i.e., within 4 h of injury) whereas of the 216 presenting with an illness, 41% [80] presented within 2–7 days of the onset of the illness.
Woolfenden et al., 2000 [75]	Australia	Explore the parental attitudes, perceptions and beliefs that play a role in the use of a tertiary paediatric emergency department (PED) when a child has a non-urgent illness.	Qualitative; Interviews; Thematic analysis	Parents of children with non-urgent illnesses recruited in the waiting room of a tertiary PED	Parents used their own system of triage to choose the appropriate service for their sick child. The perceived expertise of the tertiary PED, access and parental expectations all appeared to be major factors in parental use of a PED.
Zandieh et al., 2009 [76]	USA	Determine important predictors of why parents seek care for their children at a pediatric emergency department (ED) compared to their child’s primary care provider’s (PCP’s) walk-in clinic.	Quantitative; Cross-sectional survey; Statistics	Convenience	87 (51%) were seeking care at the ED and 83 (49%) at their child’s walk-in clinic. In logistic regression, single parenting was the strongest predictor for seeking care in the ED (OR, 5.54; 95% confidence interval [CI], 1.4Y26.9), followed by Hispanic ethnicity (OR, 4.96; 95% CI, 1.43–17.2), low parental perceptions of their child’s physical health (OR, 0 .90; 95% CI, 0.82Y0.99), controlling for number of chronic conditions, parental working status, and satisfaction with their PCP.
Zickafoose et al., 2015 [77]	USA	Assess parents’ relative preferences for different categories of enhanced access services in primary care.	Quantitative; DCE; Mixed logic model	Participants were sampled from a nationally representative online panel of individuals maintained by Knowledge Networks, a survey research firm	Parents were most likely to choose primary care offices that guaranteed same-day sick visits (coefficient, 0.57 followed by those with higher professional continuity (coefficient, 0.36 [SE, 0.03]; P < .001). Parents were also significantly more likely to choose practices with 24-h telephone advice plus non-urgent email advice (0.08 [0.04]; *P* < .05), evening hours 4 or more times a week (0.14 [0.04]; *P* < .001), and at least some hours on weekends. Parents were significantly less likely to choose practices that were closed during some weekday daytime hours or had wait times longer than 4 weeks for preventive care visits. There was very little variation in preferences among parents with different sociodemographic characteristics. Parents’ marginal willingness to travel was 14 min (95% CI, 11–16 min) for guaranteed same-day sick visits and 44 min (95% CI, 37–51 min) for an office with idealized levels of all services.
Zickafoose et al., 2013 [78]	USA	Explore (1) parents’ preferences for enhanced access services in a pediatric primary care medical home and (2) parents’ willingness to make trade-offs between enhanced access services and other aspects of primary care.	Qualitative; Semi-structured interviews; Thematic analysis	Purposive	Parents had strong preferences for certain services, such as same-day sick care appointments, and were willing to make trade-offs for high-priority services.

Immigrant and minority populations were found to be more likely to use the ED as a source of first-contact care [40, 73] with lower levels of acculturation related to even greater use of the ED in Latin American populations in the USA [73], and low abilities in the native language also associated with higher ED use in Sweden [40]. Health literacy, which has been defined as the “*skills that determine the motivation and ability of individuals to gain access to, understand and use information in ways which promote and maintain good health*” [81], was also a relevant factor [56] with lower levels of health literacy associated with greater ED use [57]. Other factors included lower income [34] and use of public health insurance programmes based on income such as Medicaid in the USA [27, 51, 61] or a lack of any health insurance [64]. In one study from Brazil, parents often utilised private healthcare as a substitute for public health services when they were unavailable, although the public health services occasionally met important needs such as paediatric-specific emergency departments not provided within the private health system [35].

While the clinical reason for attendance was not a primary focus of the current review, which sought to collate non-clinical factors, it is difficult to completely isolate the non-clinical factors from the clinical reason for attendance. Indeed, as a common childhood condition, asthma was central to a number of the included studies (*n* = 4). With regards to children with asthma, minority children were more likely to utilise urgent care compared to non-minority children in the USA when other relevant factors were controlled for including income, gender, source of usual asthma care [42] and frequency of night-time symptoms [69]. Moreover, among a pre-dominantly Latino population in an American hospital, perception of acute need was the main reason parents sought the ED for their children with asthma, however, those who use the ED do so due to barriers using primary care for unscheduled appointments [54].

Parental-specific factors were also identified in the review as influencing where first-contact care was sought. For instance, mothers who reported as being in the highest tertile of depression were more likely to bring their child to the ED rather than the GP [30] and younger parental age which was associated with a greater likelihood to seek care at the ED [34]. In a study exploring care seeking in lone parents in the UK with a partner on active duty abroad, a lack of support at home increased the likelihood that they would seek care when their child was unwell [32]. This latter finding is analogous to other studies which found that being a single-parent was a risk factor for higher ED use for non-urgent conditions [61, 76], such that parents with limited social support were more frequent ED attenders [37] and living in low income areas made the ED a more convenient choice for stressed families [38].

Pre-disposing factors are multi-faceted, inter-related and can be difficult to isolate from systems factors that also affect healthcare-seeking behaviour at unscheduled services. However, given their influence in care-seeking behaviour, it is important to report them in the present review.

### Factors that influence decision to attend and choice of unscheduled healthcare

The following factors emerged from the data as directly influencing parental choice of attendance at ED, primary care and out of hour’s services. Table 4 outlines these results from the review.

#### The need for reassurance

The need for reassurance featured heavily as a common reason parents seek healthcare at the ED. Specifically, parents wanted reassurance that their child’s illness is not serious or will not become more urgent, while also seeking guidance on how to manage the condition [25, 37, 41, 56, 60, 82]. Reassurance and seeking guidance on how to manage specific conditions such as respiratory tract infections (RTI) [49, 71] and for general illnesses [66] also factored into the decision of parents choosing to attend their GP. Parental self-efficacy and ability to cope tended to increase with more parental experience due to having other children, and this in turn influenced the decision to consult healthcare for RTIs [49, 50]. Moreover, while social pressures to seek care for their children in order to be seen to be ‘doing the right thing’ as a parent [66] was also related to care seeking, fear of wasting the doctor’s time for a minor illness was perceived as a barrier to seeking primary care [49].

#### Shorter waiting time and after-hours access to the ED compared to primary care

A number of the included studies (*n* = 9) concluded that shorter waiting times, availability and accessibility of the ED after hours was a significant factor in parents’ decision to attend the ED [25–27, 31, 33, 45, 64, 70, 75]. In a further qualitative study, parents stated that they wanted to avoid double waiting if they were sent to the ED by the GP [46].

#### Timely access to the GP (both during normal working times and after hours)

The unavailability of a timely appointment with the GP also increased the likelihood that parents would seek care in the ED [28, 41]. Moreover, one study that explored return visits to the ED stated that a lack of availability of GP appointments led to return visits to the ED [29]. Another common issue regarding ED attendance was an inability to contact the GP by phone prior to ED attendance, with between half and three-quarters of parents attempting to contact the GP prior to presenting at the ED [26, 43, 47, 48, 59, 60, 74, 83]. There were no differences in SES for parents who attempted to make contact with the GP prior to attending ED [48]. Parents were more likely to attend the ED without referral from the GP during evening and weekends [36].

#### Satisfaction with GP

A positive relationship with the GP, overall clinic environment and friendly staff were associated with choosing primary care as the first contact for care [51]. Problems with primary care include poor communication and general dissatisfaction with their GP [27, 33], however, one study did not find that problems with primary care was a clear motivator for parents to choose the ED over the GP for non-urgent conditions [38]. While the problems with primary care contributed to greater ED attendance rates, on balance, two studies found that convenience and satisfaction with primary care increased the likelihood that parents would seek care from their GP [51, 66].

#### Convenience

While only a small number of papers explored the reasons that parents choose their GP or primary care provider as the first contact for care, many of the reasons for choosing primary care were similar to those for choosing ED. For instance, in a study comparing parents who chose the paediatric ED with those who would choose primary care [59], it was found that parents chose primary care because it was more convenient [53], they would be seen quicker and they could get in touch more easily. Indeed, convenience and appointment availability [51] and travel time and same day appointments [77, 78] were also identified as important factors.

Five studies found that proximity or location of the ED was a factor in parents’ decision to utilise this service [25, 31, 39, 67] with city-dwellers from lower socio-economic areas more likely to use the ED [83] as they live closer to the hospital. With regards to primary care, a discrete choice experiment (DCE) of preferences for enhanced access to a primary care (in the medical home model) found that parents were willing to spend an additional 14 min traveling for a same day visit [77].

#### Perception of higher quality care in the ED

One of the most commonly occurring reasons for parents to choose the ED as a source of unscheduled healthcare for their children was the perception that higher quality care is available in EDs [25, 33, 39, 53, 58, 64, 72, 75, 84]. This finding also relates to the diagnostic and other equipment typically available in a hospital setting but not in a GP practice. Parents stated that they preferred the ED as diagnostics such as blood tests and X-rays can be carried out immediately, and they believed their child would get a more thorough examination by doctors in a paediatric ED [25, 28, 33]. Parental trust in ED doctors was also an important factor when seeking care for injuries [44]. On balance, dissatisfaction with the ED, including disappointment with medical staff, care and information, increased the likelihood of a return visit to the ED in one study [29].

#### Perceived urgency or severity of illness

Parents’ perception of the urgency or severity of their child’s illness also played an important role in parents’ decision to consult the ED [45, 52, 53, 56, 60, 68, 74, 75, 82, 84]. An increased perception of an illness as being urgent was also found to be associated with differing levels of health literacy as parents – those with low health literacy were more likely to seek care immediately [56]. The perceived severity of a child’s condition decreased with the age of the child, as parents’ perceived children less than 1 year old to have conditions requiring more urgent care than older children [60].

#### ED compared with out-of-hours services

Four studies explored parents’ decision making when choosing to attend out-of-hours’ healthcare, with a particular focus on use of these services in comparison to the ED. For instance, two studies [62, 63] explored the factors that influenced preferences for out-of-hours’ care and found that waiting times and receiving an adequate explanation or reassurance about their child’s illness were two of the most important factors when choosing where to seek care after hours. However, experience was also a key factor and parents who had used a GP cooperative previously were more likely to do so again [63]. Waiting times and convenience were also key factors in the use of out-of- hours’ services, and patients with knowledge of the system were more likely to utilise it [55]. Nevertheless, while ED care was still the preferred location of care for parents, whether the doctor seemed to listen was the most important attribute when evaluating different models of out-of-hours care [65].

## Discussion

The present systematic review sought to examine the non-medical factors that influence parents’ decision making when seeking unscheduled healthcare for their child. From a patient perspective, the boundaries between unscheduled health services are less pronounced than they may seem from the perspective of health providers [19]. The current review adopted this approach by extracting data related to different types of unscheduled health services (namely primary care, the emergency department and out-of-hours services) and synthesising them as one system of healthcare. Strengthening first contact care is a key focus for paediatric healthcare in Europe [14] and it is important to examine how factors influencing utilisation of these services relate to and interact with one another, and the contexts in which certain behaviours occur.

### Initiating help-seeking behaviour: perception of urgency and the need for reassurance

A parents’ decision regarding “when” to seek healthcare for their child can be influenced by the perception that their child’s condition or illness was urgent and the need for reassurance or an explanation from a healthcare professional. This initial decision to seek care is rarely a straightforward one for parents and anxiety can be heightened when making decisions for others, such as young children who may struggle to communicate their symptoms [85]. A common focus of the studies included in the review was the use of the ED for low-acuity or non-urgent conditions, however, it is difficult to synthesise these findings as there was considerable heterogeneity in how non-urgent or low-acuity conditions were defined by researchers. This is reflected in the literature where there is a lack of agreement among ED physicians on how to define an “inappropriate” visit to the ED [85], and while they recognise that certain illnesses and conditions can be treated elsewhere, they do not always consider such visits to be problematic [86]. In the present review, parents did take the appropriateness of an ED visit into account [49] and indeed, it was clear that parents do make attempts to contact a GP ahead of attending the ED [40, 43]. Navigating “appropriate” use of the unscheduled healthcare system can be challenging for patients [19] and a more nuanced understanding of how parents make sense of illness and urgency of care seeking is required.

Health literacy was found to influence a parents’ perception of urgency and in turn, their choice of service. Interventions to improve parental health literacy can reduce presentations to the emergency department [87] as parents’ understanding of health and management of illness may reduce their need to seek care elsewhere. Moreover, chronic conditions such as asthma or disability place greater caring demands on parents which further disadvantages those with lower health literacy [87]. Experiences such as being a lone parent increased care seeking [32] and non-urgent use of the ED [61]. Once a parent has decided to seek healthcare for their child, they will access care in the quickest and most convenient place at any given time. In order to enhance access and facilitate patient contact with the health service in a way that will result in the best health outcomes, we need to understand the decision making process regarding “where” care is sought, and therefore inform the design of accessible first contact services for unscheduled care.

### The choice of unscheduled health service: practical considerations and the relationship with your GP

The review identified a number a pre-disposing factors that can influence where parents choose to seek unscheduled healthcare for their child. For instance, socioeconomically disadvantaged and immigrant parents were typically more likely to seek healthcare in the ED, with this effect observed in Australia, Brazil, and the USA in the articles in the current review [34, 35, 51, 61, 64, 69, 73]. However, at the core of access and availability to unscheduled healthcare are practical issues and concerns that families must consider when seeking healthcare, and it is important to recognise that constraints within a health system can limit the options for some parents regarding where to initiate contact with the health service. For instance, the unavailability of appointments with the GP within a reasonable timeframe (e.g., within 24 h) causes parents to seek healthcare in the ED [26], and the times of available appointments can also be restrictive as they are typically during standard working hours. Parents also perceive access to diagnostic tests and the specialist equipment available in a hospital as important [25], or may go straight to the ED to avoid having to “wait twice” if they think they will be sent on to the ED by their GP anyway [46]. Due to the temporal structure of primary care and the limited diagnostic tests available, attendances at EDs for non-urgent conditions are often inevitable, therefore, strategies for reducing “inappropriate” visits to the ED could instead focus on investment in primary care to take the pressure of EDs and provide greater care in the community.

While these pragmatic concerns can impact where parents seek unscheduled healthcare, the relationship between a GP and a parent or family was also found to be an important factor when parents were considering the option of attending primary care or the ED. Socioeconomic vulnerabilities can be further exacerbated by differing experiences of primary care and other healthcare services. For example, in a study from Hong Kong, which has a primary care system dominated by private healthcare, patients with higher incomes and private health insurance reported favourable experiences in primary care [88]. In the present review, an unsatisfactory relationship with your GP was related to higher use of the ED [33] and evidence suggests that families with high income and education were more likely to report a positive relationship with their child’s GP, and reported greater involvement in decision making around their child’s health [89]. Moreover, another study in the review reported that parents with greater health literacy placed a high value on a close relationship with their GP and were willing to wait longer for an appointment [56].

### Recommendations for future research and implications for policy

The studies included in the systematic review each focused on a specific health service or services that fell within the scope of unscheduled healthcare, however, none of the studies examined parent’s utilisation or preferences for first-contact healthcare as a single service with multiple shared characteristics and entry points. It is clear from the current findings that while parents may utilise different health services as a source of unscheduled healthcare, they are using these different services for similar reasons and also operating within constraints that exist in their health system. Furthermore, the persistent framing of non-urgent, low-acuity or ‘unnecessary’ visits to the ED as problematic behaviour on the part of parents may be shifting focus away from the challenges in the system of unscheduled healthcare that result in this behaviour. While targeted interventions that improve health literacy can reduce presentations to the ED [87] and educate parents on management of minor childhood illnesses, understanding parents’ behaviour as part of a system of unscheduled healthcare should be an important priority for future research. Such research can inform policy and practice in this area by identifying opportunities for intervention that are responsive to parents’ behaviour and needs. Finally, the impact of the COVID-19 pandemic on paediatric attendance to EDs has been noted [80, 90], and it is likely that this has impacted parental decision-making when seeking unscheduled care more broadly, however, further research is needed to understand decision-making during the pandemic [91].

### Limitations

The review sought to include sources of unscheduled healthcare where patients are required to attend in person, however, other forms of unscheduled support and advice are available in some jurisdictions. For instance, pharmacists often provide advice to patients, however, the evidence around the effectiveness of this advice requires further study [92]. Some health systems provide telephone advice services where patients can speak with healthcare professionals for advice on whether to attend the ED or to receive guidance on how to manage a condition, although the evidence around these service is unclear [1]. On a related note, much of the literature made reference to parents phoning their GP for advice ahead of attending the ED, however, the outcomes of the calls were not consistently reported and it is difficult to ascertain the impact this had on attendance. Chronic conditions such as asthma will increase attendance at unscheduled services, however, this attendance is still heavily influenced by the issues brought up in the review. Further limitations of the study were the lack of focus on the clinical reason for a visit as we sought to examine the non-clinical reasons for attendance at unscheduled healthcare and the exclusion of non-English articles.

## Conclusion

The present review and narrative synthesis identified a number of factors that can influence parental preferences and decision making when seeking unscheduled paediatric healthcare. Parental decisions on when and where to seek unscheduled healthcare for their children are not made in a vacuum as parents weigh up the options in front of them, utilise prior experiences and make the most appropriate decision in any given context. While a strong system of primary care has been associated with more positive population health outcomes [93], access issues that are faced by subsections of the population and the practical considerations of parents are substantial limitations that need to be addressed. Policy and planning initiatives do not always reflect how patients negotiate the health system as a single entity with numerous entry points [19, 85]. Altering patients’ behaviour through public health initiatives that seek to improve, for instance, health literacy [87] or reducing emergency hospital admissions through preventative primary care [9] requires an understanding of the relative importance of factors that influence behaviour and decision making, and the interactions between these factors.

## Supplementary information

**Additional file 1.** Table 1. Full electronic search of PubMed.

**Additional file 2 **Table 2**.** Quality Assessment Scores using the Mixed Methods Assessment Tool (MMAT).

## Data Availability

All data analysed during this study are included in this published article.

## Abbreviations

GP: General Practitioners
ED: Emergency Department
SES: Socioeconomic Status
