# Supplementary material for: Factors that influence family and parental preferences and decision making for unscheduled paediatric healthcare – systematic review
Source: BMC Health Serv Res. 2020 Jul 17;20:663. doi: 10.1186/s12913-020-05527-5 (PMC7366445; doi:10.1186/s12913-020-05527-5)
Supplement: Supplementary file 1 — Additional file 1. Table 1. Full electronic search of PubMed. [file 12913_2020_5527_MOESM1_ESM.docx]

| **Supplementary Table 1: Full electronic search of PubMed** | | |
| --- | --- | --- |
| **Database** | **Search Options** | **Indexed Terms** |
| PubMed | Limiters: English,  Publication date: 2000/01/01-12/03/2019 | (((((((Child*[Title/Abstract]) OR paediatric[Title/Abstract]) OR pediatric[Title/Abstract]) OR Infant[Title/Abstract]) OR adolescent[Title/Abstract])) AND ((((("Parent* preferences"[Title/Abstract]) OR choice*[Title/Abstract]) OR "decision making"[Title/Abstract]) OR "Family preferences"[Title/Abstract]) OR Reasons[Title/Abstract])) AND ((((((((("primary care"[Title/Abstract]) OR "general practice"[Title/Abstract]) OR "family physician"[Title/Abstract]) OR "emergency care"[Title/Abstract]) OR "emergency department"[Title/Abstract]) OR "out-of-hours"[Title/Abstract]) OR "Practitioner Cooperative"[Title/Abstract]) OR "after hours"[Title/Abstract]) OR "urgent care cent*"[Title/Abstract]) |
