# Supplementary material for: Factors that influence family and parental preferences and decision making for unscheduled paediatric healthcare – systematic review
Source: BMC Health Serv Res. 2020 Jul 17;20:663. doi: 10.1186/s12913-020-05527-5 (PMC7366445; doi:10.1186/s12913-020-05527-5)
Supplement: Supplementary file 2 — Additional file 2 Table 2. Quality Assessment Scores using the Mixed Methods Assessment Tool (MMAT). [file 12913_2020_5527_MOESM2_ESM.docx]

**Supplementary Table 2. Quality Assessment Scores using the Mixed Methods Assessment Tool (MMAT)**

| Study | Screening Questions | | Qualitative | | | | Quantitative | | | | | | | | | | | | Mixed Methods | | |  |  |  |  |  |  |  |  |  |  |  |  |  |  |  |  |  |  |  |  |  |
| --- | --- | --- | --- | --- | --- | --- | --- | --- | --- | --- | --- | --- | --- | --- | --- | --- | --- | --- | --- | --- | --- | --- | --- | --- | --- | --- | --- | --- | --- | --- | --- | --- | --- | --- | --- | --- | --- | --- | --- | --- | --- | --- |
|  |  |  |  |  |  |  | Randomised Controlled trial | | | | Non-Randomised Controlled Trial | | | | Quantitative Descriptive | | | |  |  |  |  |  |  |  |  |  |  |  |  |  |  |  |  |  |  |  |  |  |  |  |  |
|  | S1 | S2 | 1.1 | 1.2 | 1.3 | 1.4 | 2.1 | 2.2 | 2.3 | 2.4 | 3.1 | 3.2 | 3.3 | 3.4 | 4.1 | 4.2 | 4.3 | 4.4 | 5.1 | 5.2 | 5.3 |  |  |  |  |  |  |  |  |  |  |  |  |  |  |  |  |  |  |  |  |  |
| Albrecht et al, 2017 | y | y | y | y | y | n |  |  |  |  |  |  |  |  |  |  |  |  |  |  |  |  |  |  |  |  |  |  |  |  |  |  |  |  |  |  |  |  |  |  |  |  |
| Augustine et al, 2016 | y | y | y | y | n | n |  |  |  |  |  |  |  |  |  |  |  |  |  |  |  |  |  |  |  |  |  |  |  |  |  |  |  |  |  |  |  |  |  |  |  |  |
| Bartlett et al, 2001 | y | y |  |  |  |  |  |  |  |  |  |  |  |  | y | y | y | c |  |  |  |  |  |  |  |  |  |  |  |  |  |  |  |  |  |  |  |  |  |  |  |  |
| Benahmed et al, 2012 | y | y |  |  |  |  |  |  |  |  |  |  |  |  | y | y | c | y |  |  |  |  |  |  |  |  |  |  |  |  |  |  |  |  |  |  |  |  |  |  |  |  |
| Bernthal et al, 2017 | y | y | y | y | y | y |  |  |  |  |  |  |  |  |  |  |  |  |  |  |  |  |  |  |  |  |  |  |  |  |  |  |  |  |  |  |  |  |  |  |  |  |
| Berry et al, 2008 | y | y | y | y | y | c |  |  |  |  |  |  |  |  |  |  |  |  |  |  |  |  |  |  |  |  |  |  |  |  |  |  |  |  |  |  |  |  |  |  |  |  |
| Bingham et al, 2015 | y | y |  |  |  |  |  |  |  |  |  |  |  |  | y | n | y | y |  |  |  |  |  |  |  |  |  |  |  |  |  |  |  |  |  |  |  |  |  |  |  |  |
| Buboltz et al, 2015 | y | y | y | y | y | n |  |  |  |  |  |  |  |  |  |  |  |  |  |  |  |  |  |  |  |  |  |  |  |  |  |  |  |  |  |  |  |  |  |  |  |  |
| Burokienė et al, 2017 | y | y | y | n | y | c |  |  |  |  |  |  |  |  |  |  |  |  |  |  |  |  |  |  |  |  |  |  |  |  |  |  |  |  |  |  |  |  |  |  |  |  |
| Cabey et al, 2018 | y | y | y | y | y | c |  |  |  |  |  |  |  |  |  |  |  |  |  |  |  |  |  |  |  |  |  |  |  |  |  |  |  |  |  |  |  |  |  |  |  |  |
| Chin et al, 2006 | y | y | y | c | y | c |  |  |  |  |  |  |  |  |  |  |  |  |  |  |  |  |  |  |  |  |  |  |  |  |  |  |  |  |  |  |  |  |  |  |  |  |
| Cooper et al, 2003 | y | y |  |  |  |  |  |  |  |  |  |  |  |  | y | c | c | y |  |  |  |  |  |  |  |  |  |  |  |  |  |  |  |  |  |  |  |  |  |  |  |  |
| Ellbrandt et al, 2018 | y | y |  |  |  |  |  |  |  |  |  |  |  |  | c | c | y | y |  |  |  |  |  |  |  |  |  |  |  |  |  |  |  |  |  |  |  |  |  |  |  |  |
| Fieldston et al, 2012 | y | y | y | c | y | c |  |  |  |  |  |  |  |  |  |  |  |  |  |  |  |  |  |  |  |  |  |  |  |  |  |  |  |  |  |  |  |  |  |  |  |  |
| Fredrickson et al, 2004 | y | y | y | c | y | n |  |  |  |  |  |  |  |  | y | y | y | c | y | n | c |  |  |  |  |  |  |  |  |  |  |  |  |  |  |  |  |  |  |  |  |  |
| Freed et al, 2016 | Y | Y |  |  |  |  |  |  |  |  |  |  |  |  | y | y | y | y |  |  |  |  |  |  |  |  |  |  |  |  |  |  |  |  |  |  |  |  |  |  |  |  |
| Gafforini et al, 2016 | Y | Y |  |  |  |  |  |  |  |  |  |  |  |  | y | c | y | c |  |  |  |  |  |  |  |  |  |  |  |  |  |  |  |  |  |  |  |  |  |  |  |  |
| Grant et al, 2010 | Y | Y | y | y | n | n |  |  |  |  |  |  |  |  | c | y | c | y | y | y | n |  |  |  |  |  |  |  |  |  |  |  |  |  |  |  |  |  |  |  |  |  |
| Grigg et al, 2013 | Y | Y | y | y | c | c |  |  |  |  |  |  |  |  |  |  |  |  |  |  |  |  |  |  |  |  |  |  |  |  |  |  |  |  |  |  |  |  |  |  |  |  |
| Guttman et al, 2003 | Y | Y | y | y | y | c |  |  |  |  |  |  |  |  |  |  |  |  |  |  |  |  |  |  |  |  |  |  |  |  |  |  |  |  |  |  |  |  |  |  |  |  |
| Harrold et al, 2018 | Y | Y |  |  |  |  |  |  |  |  |  |  |  |  | y | y | y | y |  |  |  |  |  |  |  |  |  |  |  |  |  |  |  |  |  |  |  |  |  |  |  |  |
| Hendry et al, 2004 | Y | Y |  |  |  |  |  |  |  |  |  |  |  |  | y | y | y | y |  |  |  |  |  |  |  |  |  |  |  |  |  |  |  |  |  |  |  |  |  |  |  |  |
| Ingram et al, 2013 | Y | Y | y | y | y | n |  |  |  |  |  |  |  |  |  |  |  |  |  |  |  |  |  |  |  |  |  |  |  |  |  |  |  |  |  |  |  |  |  |  |  |  |
| Janicke et al, 2003 | Y | Y |  |  |  |  |  |  |  |  |  |  |  |  | y | c | y | c |  |  |  |  |  |  |  |  |  |  |  |  |  |  |  |  |  |  |  |  |  |  |  |  |
| Klein et al, 2011 | Y | Y | y | y | y | n |  |  |  |  |  |  |  |  | y | y | y | c | y | c | n |  |  |  |  |  |  |  |  |  |  |  |  |  |  |  |  |  |  |  |  |  |
| Kua et al, 2016 | Y | Y | y | y | y | c |  |  |  |  |  |  |  |  |  |  |  |  |  |  |  |  |  |  |  |  |  |  |  |  |  |  |  |  |  |  |  |  |  |  |  |  |
| Kubicek et al, 2012 | Y | Y |  |  |  |  |  |  |  |  |  |  |  |  | y | y | n | c |  |  |  |  |  |  |  |  |  |  |  |  |  |  |  |  |  |  |  |  |  |  |  |  |
| Lara et al, 2003 | Y | Y |  |  |  |  |  |  |  |  |  |  |  |  | y | c | c | n |  |  |  |  |  |  |  |  |  |  |  |  |  |  |  |  |  |  |  |  |  |  |  |  |
| Lass et al, 2018 | Y | Y | y | y | y | y |  |  |  |  |  |  |  |  |  |  |  |  |  |  |  |  |  |  |  |  |  |  |  |  |  |  |  |  |  |  |  |  |  |  |  |  |
| Long et al, 2018 | Y | Y |  |  |  |  |  |  |  |  |  |  |  |  | y | c | y | n |  |  |  |  |  |  |  |  |  |  |  |  |  |  |  |  |  |  |  |  |  |  |  |  |
| May et al, 2018 | Y | Y | y | y | y | c |  |  |  |  |  |  |  |  |  |  |  |  |  |  |  |  |  |  |  |  |  |  |  |  |  |  |  |  |  |  |  |  |  |  |  |  |
| Morrison et al, 2014 | Y | Y |  |  |  |  |  |  |  |  |  |  |  |  | y | c | y | y |  |  |  |  |  |  |  |  |  |  |  |  |  |  |  |  |  |  |  |  |  |  |  |  |
| Mostajer et al, 2016 | Y | Y | y | y | y | y |  |  |  |  |  |  |  |  |  |  |  |  |  |  |  |  |  |  |  |  |  |  |  |  |  |  |  |  |  |  |  |  |  |  |  |  |
| Newcomb et al, 2005 | Y | Y |  |  |  |  |  |  |  |  |  |  |  |  | y | c | y | y |  |  |  |  |  |  |  |  |  |  |  |  |  |  |  |  |  |  |  |  |  |  |  |  |
| Nokoff et al, 2014 | Y | Y |  |  |  |  |  |  |  |  |  |  |  |  | y | c | n | y |  |  |  |  |  |  |  |  |  |  |  |  |  |  |  |  |  |  |  |  |  |  |  |  |
| Ogilivie et al, 2016 | Y | Y |  |  |  |  |  |  |  |  |  |  |  |  | y | c | y | y |  |  |  |  |  |  |  |  |  |  |  |  |  |  |  |  |  |  |  |  |  |  |  |  |
| Pethe et al, 2019 | Y | Y |  |  |  |  |  |  |  |  |  |  |  |  | y | c | n | y |  |  |  |  |  |  |  |  |  |  |  |  |  |  |  |  |  |  |  |  |  |  |  |  |
| Phelps et al, 2000 | Y | Y |  |  |  |  |  |  |  |  |  |  |  |  | y | c | y | c |  |  |  |  |  |  |  |  |  |  |  |  |  |  |  |  |  |  |  |  |  |  |  |  |
| Philips et al, 2012 | Y | Y |  |  |  |  |  |  |  |  |  |  |  |  | y | c | y | c |  |  |  |  |  |  |  |  |  |  |  |  |  |  |  |  |  |  |  |  |  |  |  |  |
| Philips et al, 2010 | Y | Y |  |  |  |  |  |  |  |  |  |  |  |  | y | c | y | n |  |  |  |  |  |  |  |  |  |  |  |  |  |  |  |  |  |  |  |  |  |  |  |  |
| Salami et al, 2012 | Y | Y |  |  |  |  |  |  |  |  |  |  |  |  | y | c | y | y |  |  |  |  |  |  |  |  |  |  |  |  |  |  |  |  |  |  |  |  |  |  |  |  |
| Scott et al, 2003 | Y | Y |  |  |  |  |  |  |  |  |  |  |  |  | y | c | y | y |  |  |  |  |  |  |  |  |  |  |  |  |  |  |  |  |  |  |  |  |  |  |  |  |
| Sharma et al, 2014 | Y | Y | y | c | y | n |  |  |  |  |  |  |  |  |  |  |  |  |  |  |  |  |  |  |  |  |  |  |  |  |  |  |  |  |  |  |  |  |  |  |  |  |
| Siminski et al, 2008 | Y | Y |  |  |  |  |  |  |  |  |  |  |  |  | y | c | n | y |  |  |  |  |  |  |  |  |  |  |  |  |  |  |  |  |  |  |  |  |  |  |  |  |
| Smith et al, 2015 | Y | Y |  |  |  |  |  |  |  |  |  |  |  |  | y | c | n | y |  |  |  |  |  |  |  |  |  |  |  |  |  |  |  |  |  |  |  |  |  |  |  |  |
| Stanley et al, 2007 | Y | Y |  |  |  |  |  |  |  |  |  |  |  |  | y | c | y | y |  |  |  |  |  |  |  |  |  |  |  |  |  |  |  |  |  |  |  |  |  |  |  |  |
| Stingone et al, 2005 | Y | Y |  |  |  |  |  |  |  |  |  |  |  |  | y | y | y | y |  |  |  |  |  |  |  |  |  |  |  |  |  |  |  |  |  |  |  |  |  |  |  |  |
| Stockwell et al, 2011 | Y | Y |  |  |  |  |  |  |  |  |  |  |  |  | y | c | y | c |  |  |  |  |  |  |  |  |  |  |  |  |  |  |  |  |  |  |  |  |  |  |  |  |
| Stoddart et al, 2006 | Y | Y | y | y | y | y |  |  |  |  |  |  |  |  |  |  |  |  |  |  |  |  |  |  |  |  |  |  |  |  |  |  |  |  |  |  |  |  |  |  |  |  |
| Turbitt et al, 2016 | Y | Y |  |  |  |  |  |  |  |  |  |  |  |  | y | c | y | c |  |  |  |  |  |  |  |  |  |  |  |  |  |  |  |  |  |  |  |  |  |  |  |  |
| Vaughn et al, 2012 | Y | Y |  |  |  |  |  |  |  |  |  |  |  |  | y | n | y | c |  |  |  |  |  |  |  |  |  |  |  |  |  |  |  |  |  |  |  |  |  |  |  |  |
| Williams et al, 2009 | Y | Y |  |  |  |  |  |  |  |  |  |  |  |  | y | y | y | y |  |  |  |  |  |  |  |  |  |  |  |  |  |  |  |  |  |  |  |  |  |  |  |  |
| Woolfenden et al, 2000 | Y | Y | y | y | n | n |  |  |  |  |  |  |  |  |  |  |  |  |  |  |  |  |  |  |  |  |  |  |  |  |  |  |  |  |  |  |  |  |  |  |  |  |
| Zandieh et al, 2009 | Y | Y |  |  |  |  |  |  |  |  |  |  |  |  | y | c | y | n |  |  |  |  |  |  |  |  |  |  |  |  |  |  |  |  |  |  |  |  |  |  |  |  |
| Zickafoose et al, 2015 | Y | Y | y | y | n | n |  |  |  |  |  |  |  |  |  |  |  |  |  |  |  |  |  |  |  |  |  |  |  |  |  |  |  |  |  |  |  |  |  |  |  |  |
| Zickafoose et al, 2013 | Y | Y |  |  |  |  |  |  |  |  |  |  |  |  | y | y | y | c |  |  |  |  |  |  |  |  |  |  |  |  |  |  |  |  |  |  |  |  |  |  |  |  |

Note: y = yes; n = no; c = can’t tell
